# Influence of Climate on Google Internet Searches for Pruritus Across 16 German Cities: Retrospective Analysis

**DOI:** 10.2196/13739

**Published:** 2019-07-12

**Authors:** Linda Tizek, Maximilian Schielein, Melvin Rüth, Sonja Ständer, Manuel Pedro Pereira, Bernadette Eberlein, Tilo Biedermann, Alexander Zink

**Affiliations:** 1 Department of Dermatology and Allergy School of Medicine Technical University of Munich Munich Germany; 2 The Institute for Medical Information Processing, Biometry, and Epidemiology Ludwig Maximilian University Munich Munich Germany; 3 Center for Chronic Pruritus Department of Dermatology University Hospital Münster Muenster Germany

**Keywords:** pruritus, Internet, informatics, environment, weather, retrospective studies

## Abstract

**Background:**

The burden of pruritus is high, especially among patients with dermatologic diseases. Identifying trends in pruritus burden and people’s medical needs is challenging, since not all affected people consult a physician.

**Objective:**

The purpose of this study was to investigate pruritus search behavior trends in Germany and identify associations with weather factors.

**Methods:**

Google AdWords Keyword Planner was used to quantify pruritus-related search queries in 16 German cities from August 2014 to July 2018. All identified keywords were qualitatively categorized and pruritus-related terms were descriptively analyzed. The number of search queries per 100,000 inhabitants of each city was compared to environmental factors such as temperature, humidity, particulate matter 10 micrometers or less in diameter (PM10), and sunshine duration to investigate potential correlations.

**Results:**

We included 1150 pruritus-related keywords, which resulted in 2,851,290 queries. “Pruritus” (n=115,680) and “anal pruritus” (n=102,390) were the most-searched-for keywords. Nearly half of all queries were related to the category *localization*, with Berlin and Munich having a comparatively high proportion of people that searched for pruritus in the genital and anal areas. People searched more frequently for information on chronic compared to acute pruritus. The most populated cities had the lowest number of queries per 100,000 inhabitants (Berlin, n=13,641; Hamburg, n=18,303; and Munich, n=21,363), while smaller cities (Kiel, n=35,027; and Freiburg, n=39,501) had the highest. Temperature had a greater effect on search query number (beta -7.94, 95% CI -10.74 to -5.15) than did PM10 (beta -5.13, 95% CI -7.04 to -3.22), humidity (beta 4.73, 95% CI 2.70 to 6.75), or sunshine duration (beta 0.66, 95% CI 0.36 to 0.97). The highest relative number of search queries occurred during the winter (ie, December to February).

**Conclusions:**

By taking into account the study results, Google data analysis helps to examine people’s search frequency, behavior, and interest across cities and regions. The results indicated a general increase in search queries during the winter as well as differences across cities located in the same region; for example, there was a decline in search volume in Saarbrucken, while there were increases in Cologne, Frankfurt, and Dortmund. In addition, the detected correlation between search volume and weather data seems to be valuable in predicting an increase in pruritus burden, since a significant association with rising humidity and sunshine duration, as well as declining temperature and PM10, was found. Accordingly, this is an unconventional and inexpensive method to identify search behavior trends and respective inhabitants’ needs.

## Introduction

Pruritus is one of the most common presenting symptoms in dermatological patients, appearing in more than 50% of patients [1,2]. In general, acute and chronic pruritus occurrences are most prevalent among people affected by scabies (up to 100%) [3], atopic dermatitis (83.3%-91.0%) [2,4,5], and psoriasis (48.6%-84.0%) [5,6]. Epidemiological studies have reported point prevalence rates in the general population ranging from 3.6% to 8.4% for acute pruritus [1,5] and 13.5% to 31.2% for chronic pruritus (ie, lasting >6 weeks) [2,7,8]. Prevalence has been observed to increase with age [9].

Pruritus can have a great impact on quality of life [10-12]. There is a higher prevalence of suicidal ideation and depression among people with severe pruritus [11,13,14]. Treatment, especially for chronic pruritus, can be very challenging owing to the diversity of underlying diseases [15,16], subjective sensations, and varying individual patient needs [17,18]. There are various scales for measuring symptom intensity and severity [19]. Pruritus is also measured using patient-oriented measurements (eg, the patient benefit index) according to the individual needs and desires of patients with respect to therapeutic outcomes [20]. A study that used the patient benefit index showed that 83.9% of patients with psoriasis considered the reduction of pruritus to be a treatment goal [21]. However, people with less severe pruritus, localized manifestations, or only occasional itching might not seek professional health care; thus, it is challenging to examine people’s interest in medical needs.

An unconventional method to assess people’s interest in different aspects of pruritus outside the medical setting is to analyze the volume of Internet searches for “pruritus” and accompanying expressions, since the Internet is a commonly used source of health information [22,23]. In Germany, 90% of residents use the Internet and 72% use it daily [24]. Approximately 57% of the German population have used the Internet to search for health-related information at least once [25] and the vast majority of this group (95%) use Google as their primary search engine [26]. As previously demonstrated, Google data analysis is valuable for detecting seasonal trends and making forecasts regarding various diseases, such as cancer, epilepsy, Ebola virus disease, or influenza [27-29]. For example, as previously demonstrated in the United States, search behavior was associated with incidence rates of various tumors (eg, skin cancer) [29] or coronary heart disease. In addition, analyzing Internet data is useful for examining Internet behavior, interest, and people’s reactions to various incidents [27]. Studies investigating Google search volume data for the term “pruritus” in Germany have demonstrated that this method provides good insight into people’s interests and needs [30,31]. However, dermatological care differs even within Germany. For example, according to the German Association of Panel Doctors (Kassenärztliche Bundesvereinigung), the supply of dermatologists (ie, the proportion of target to actual number of dermatologists as a function of regional inhabitants) was comprehensively higher in Freiburg (218.2%) and Kiel (188.1%) than in Berlin (124.1%) and Hamburg (113.3%) [32]. Besides variations in the supply of dermatologists, there are regional differences in environmental triggers, such as temperature [33-37]. Accordingly, further analysis of Google data with regard to regional differences in pruritus would be valuable. Therefore, in an effort to identify possible unmet needs, this study examined the Google search volume data of 16 German cities to investigate whether there were local differences in people’s interest in pruritus and whether external factors might have had an influence on search behavior.

## Methods

### Study Design

**Figure 1:**
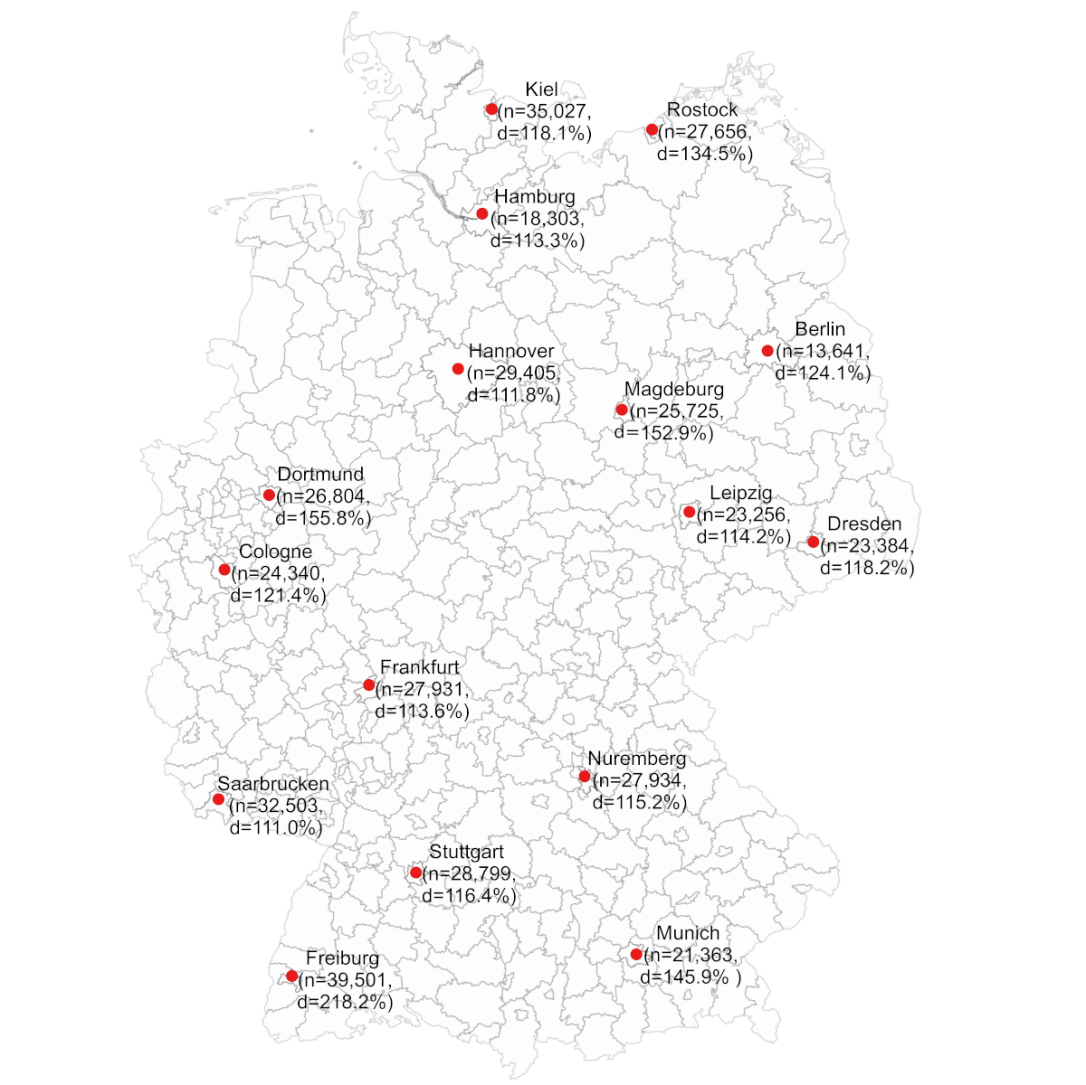
Map showing the 16 German cities for which Google search volume analysis was performed for pruritus-related terms searched from August 2014 to July 2018. n: number of search queries per 100,000 inhabitants; d: supply of dermatologists (ie, ratio of target to actual number of dermatologists with respect to population).

A retrospective longitudinal study using Google AdWords Keyword Planner was conducted to identify the Google search volume of keywords related to pruritus in 16 cities across Germany. Four cities each from Northern Germany (Hamburg, Hannover, Kiel, and Rostock), Eastern Germany (Berlin, Leipzig, Dresden, and Magdeburg), Southern Germany (Munich, Stuttgart, Nuremberg, and Freiburg), and Western Germany (Cologne, Frankfurt, Dortmund, and Saarbrucken) were chosen for a representative evaluation to determine whether there are national and geographical differences in pruritus searches within these regions. Most of the cities were chosen because they are Germany’s largest cities by population; the following 11 cities are listed in order of largest population to smallest, with their population ranking in Germany within parentheses: Berlin (1), Hamburg (2), Munich (3), Cologne (4), Frankfurt (5), Stuttgart (6), Dortmund (8), Leipzig (9), Dresden (12), Hannover (13), and Nuremberg (14). Furthermore, Kiel and Rostock were chosen to examine whether the proximity to the coast has an influence on Google search volume. The remaining cities—Freiburg, Saarbrucken, and Magdeburg—were chosen because we wanted to have a nationwide overview about various regions and they are three of the largest cities within these regions. Even though the main function of Google’s Keyword Planner is to optimize advertising, it can also be used to answer scientific questions [23,30,31]. To assess search volume within a specific field, words or phrases related to the topic are initially entered into the tool. The Keyword Planner then finds the most relevant search terms. For each identified keyword, the tool provides the monthly search volume data as estimated by Google, which are available for the last 48 months. The search volume represents the total number of searches related to selected keywords [38]. In this study, the tool was used to investigate the number of search queries associated with the German lay word for pruritus or itch (“Juckreiz”) from August 2014 to July 2018 (see Figure 1). The region and language settings were set so that the search volume data using Google products were limited solely to users in the abovementioned cities whose language preference was *German*.

Since the study was based on Google search terms, institutional review board approval was not needed and informed consent was not applicable.

### Classifications

Keywords identified by Google AdWords Keyword Planner were qualitatively analyzed. Terms that were associated with pruritus but did not explicitly include words or phrases related to pruritus (eg, “psoriasis” and “atopic dermatitis”) were initially excluded from descriptive analyses. Only when investigating a correlation between the supply of general practitioners (GPs) and search volume were these terms considered, as people consulting a GP might receive other less-specific diagnoses [36]. After qualitatively assessing all pruritus-related keywords, eight categories were formed in accordance with previous studies [30,31] to determine differences in people’s interests: *causes* (eg, “pruritus causes”), *conditions* (eg, “pruritus at night-time”), *influential factors* (eg, “allergic pruritus”), *localization* (eg, “itchy legs”), *pruritus descriptors* (eg, “strong pruritus”), *questions on pruritus* (eg, “what causes pruritus?”), and *treatment* (eg, “drugs against pruritus”); searches that did not fit any of these categories were placed in a *general* category (eg, “pruritus”). Terms matching several criteria were assigned to multiple categories. To assess differences in search behaviors across Germany, the search volume for each city was calculated in relation to its inhabitants [39] and then expressed as the number of search queries per 100,000 inhabitants (see Table 1).

**Table 1:** Number of searches per 100,000 inhabitants according to categories of pruritus-related keywords in 16 German cities from August 2014 to July 2018.

City (overall search volume)^a^ [39]	Number of inhabitants in 2016	Categories and number of searches/100,000 inhabitants, n (%)^b^							
		Causes (k^c^=94)	Conditions (k=148)	Influential factors (k=240)	Localiza­tion (k=499)	Pruritus descriptors (k=215)	Questions on pruritus (k=100)	Treatment (k=149)	General (k=109)
Berlin (N=13,641)	3,574,830	779 (5.71)	1128 (8.27)	1966 (14.41)	6119 (44.86)	1537 (11.27)	720 (5.28)	1475 (10.82)	2674 (19.60)
Hamburg (N=18,303)	1,810,438	1058 (5.78)	1665 (9.10)	2891 (15.80)	8083 (44.16)	2156 (11.78)	1012 (5.53)	1957 (10.69)	3262 (17.82)
Munich (N=21,363)	1,464,301	1209 (5.66)	1859 (8.70)	3428 (16.04)	9712 (45.46)	2496 (11.68)	1097 (5.14)	2230 (10.44)	3712 (17.37)
Cologne (N=24,340)	1,075,935	1397 (5.74)	2247 (9.23)	3988 (16.39)	10,889 (44.74)	2919 (11.99)	1357 (5.58)	2656 (10.91)	4040 (16.60)
Frankfurt (N=27,931)	736,414	1589 (5.69)	2539 (9.09)	4645 (16.63)	12,542 (44.90)	3278 (11.74)	1502 (5.38)	3073 (11.00)	4530 (16.22)
Stuttgart (N=28,799)	628,032	1705 (5.92)	2484 (8.62)	4727 (16.42)	13,122 (45.56)	3439 (11.94)	1626 (5.64)	3064 (10.64)	4657 (16.17)
Dortmund (N=26,804)	585,813	1593 (5.94)	2366 (8.83)	4350 (16.23)	12,069 (45.03)	3382 (12.62)	1726 (6.44)	3023 (11.28)	4198 (15.66)
Leipzig (N=23,256)	571,088	1392 (5.99)	1984 (8.53)	3707 (15.94)	10,093 (43.40)	2884 (12.40)	1219 (5.24)	2590 (11.14)	3977 (17.10)
Dresden (N=23,384)	547,172	1482 (6.34)	1990 (8.51)	3540 (15.14)	10,651 (45.55)	2941 (12.58)	1177 (5.03)	2601 (11.12)	3739 (15.99)
Hannover (N=29,405)	532,864	1717 (5.84)	2719 (9.25)	4851 (16.50)	12,941 (44.01)	3549 (12.07)	1687 (5.74)	3354 (11.40)	4643 (15.79)
Nuremberg (N=27,934)	511,628	1667 (5.97)	2557 (9.15)	4535 (16.23)	12,658 (45.31)	3389 (12.13)	1597 (5.72)	3145 (11.26)	4329 (15.50)
Kiel (N=35,027)	247,441	2219 (6.33)	3302 (9.43)	5508 (15.73)	15,749 (44.96)	4066 (11.61)	1908 (5.45)	3876 (11.06)	5428 (15.50)
Magdeburg (N=25,725)	238,136	1655 (6.43)	1969 (7.66)	3523 (13.70)	11,758 (45.71)	3099 (12.05)	1302 (5.06)	3141 (12.21)	4170 (16.21)
Freiburg (N=39,501)	227,590	2474 (6.26)	3203 (8.11)	6534 (16.54)	18,103 (45.83)	4302 (10.89)	2144 (5.43)	4772 (12.08)	5571 (14.10)
Rostock (N=27,656)	207,513	1711 (6.19)	2231 (8.07)	3653 (13.21)	12,867 (46.52)	3301 (11.94)	1460 (5.28)	3446 (12.46)	4274 (15.46)
Saarbrucken (N=32,503)	179,709	1898 (5.84)	2515 (7.74)	4875 (15.00)	15,442 (47.51)	3734 (11.49)	1870 (5.75)	4018 (12.36)	4646 (14.30)
Total (N=21,701)	13,138,904	1272 (5.86)	1896 (8.74)	3409 (15.71)	9759 (44.97)	2566 (11.82)	1186 (5.46)	2392 (11.02)	3676 (16.94)

^a^Overall search volume/100,000 inhabitants

^b^The cumulative percentage might be over 100% since keywords could have been attributed to multiple categories.

^c^Number of keywords.

### External Factors

Data from the Climate Data Center [34] (ie, mean monthly temperature in °C, mean humidity in %, and mean monthly sunshine duration in hours) as well as atmospheric particulate matter 10 micrometers or less in diameter (PM10) data from federal states and federal government networks (Messnetze der Bundesländer sowie des Bundes) [37] were used to determine whether there were correlations with the number of search queries within each city. In addition, the correlation between searches and the supply of dermatologists and GPs [32], as well as the respective inhabitants’ demographics (ie, mean age, proportion of female inhabitants, or proportion of nonnative German inhabitants) [39-41], was examined.

### Statistical Analysis

Descriptive data were generated for all categorized keywords. To determine differences in search volume per 100,000 inhabitants within cities and regions, one-way analysis of variance (ANOVA) was used. Pearson’s correlation coefficient was used to assess the relationship between the number of search queries and the abovementioned external factors. Additionally, a linear regression model was generated to further assess the relationship between search queries and environmental factors. Forward selection was used to generate the best-fit model. Standardized regression coefficients (beta) and 95% CIs were estimated. Stratified analyses by region were also performed. IBM SPSS Statistics for Windows, version 25.0 (IBM Corp) was used for the statistical and spatial analyses. Geodata from the German Federal Agency for Cartography and Geodesy [42] were used to determine administrative boundaries using a geographic information system, QGIS, version 2.14.22 (QGIS Development Team).

## Results

### Overview

**Figure 2:**
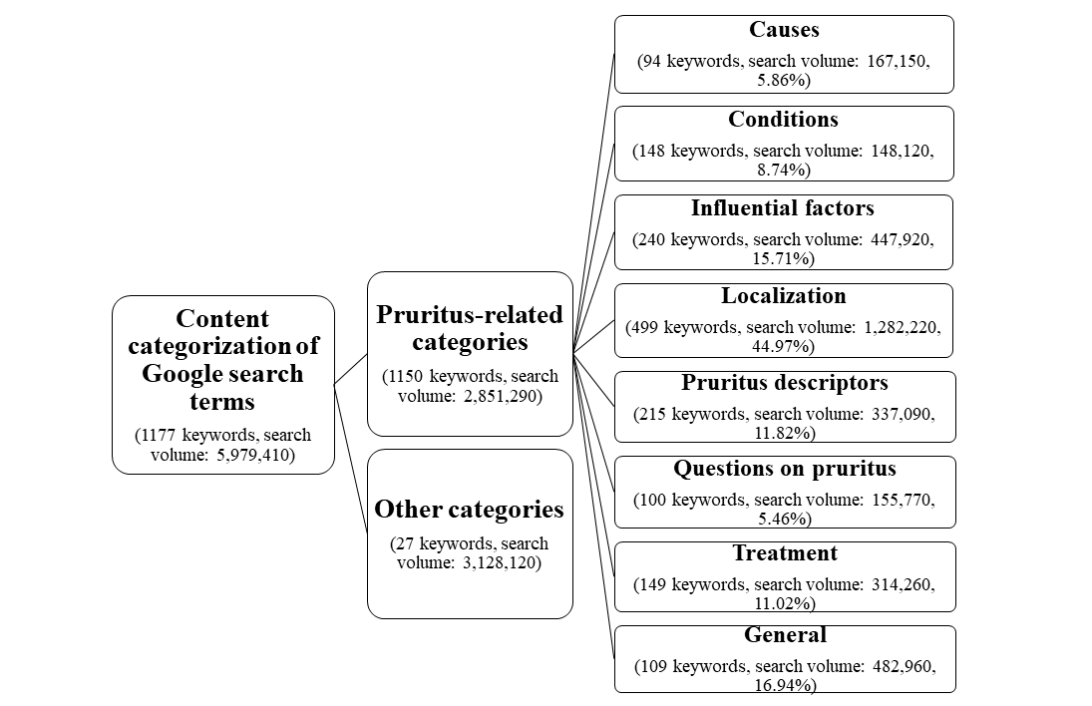
Content categorization of search terms identified by Google AdWords Keyword Planner.

In total, 1177 keywords related to the German lay word for pruritus were identified in all 16 German cities. Of these, 1150 were considered for further analyses, resulting in a search volume of 2,851,290 queries. Most of the keywords were assigned to the *localization* category (499/1150, 43.39%), whereas the smallest number of keywords were categorized as *causes* (94/1150, 8.17%) (see Figure 2). The most-searched-for keywords were “pruritus” (n=115,680), “anal pruritus” (n=102,390), “pruritus on the whole body” (n=56,660), and “itchy skin” (n=53,480) (see Multimedia Appendix 1).

### Comparison of 16 Cities Across Germany

As expected, the greatest number of absolute searches for pruritus-related keywords occurred in the most populated German cities: Berlin (n=487,650), Hamburg (n=331,360), and Munich (n=312,820). However, when adjusting the search volume by the number of inhabitants, Freiburg and Kiel had the highest number per 100,000 inhabitants, with 39,501 and 35,027 search queries, respectively (see Figure 1). The analyses showed that Berlin had a significantly lower number of search queries per 100,000 inhabitants (n=13,641) than all other cities except for Hamburg (n=18,303, *P*=.99) and Munich (n=21,363, *P*=.05). Overall, in Eastern Germany, the number of search queries (86,006 searches/100,000 inhabitants) was lower compared to Northern Germany (110,391 searches/100,000 inhabitants, *P*<.001), Southern Germany (117,597 searches/100,000 inhabitants, *P*<.001), and Western Germany (111,578 searches/100,000 inhabitants, *P*<.001).

About 43.4%-47.5% of all queries were related to the body parts affected by pruritus, meaning that *localization* was the category with the highest relative number of searches: 9759 searches per 100,000 inhabitants. Especially in Berlin and Munich, a high proportion of people (844/4103 [20.57%] searches/100,000 inhabitants and 1311/6519 [20.11%] searches/100,000 inhabitants, respectively) searched for pruritus in the genital or anal areas, while, overall, approximately 11.55% of *localization*-related keywords focused on these areas. A total of 2566 searches per 100,000 inhabitants included more specific *pruritus descriptors*, such as “constant” (457/2566 [17.81%] searches/100,000 inhabitants). In Berlin, the number of searches per 100,000 inhabitants for keywords including information on *pruritus descriptors* only was 1537, while more than 4000 searches were registered in Kiel and Freiburg. In general, people searched more frequently for information on chronic pruritus (80/2566 [3.12%] searches/100,000 inhabitants) compared to acute pruritus (17/2566 [0.66%] searches/100,000 inhabitants). The most-searched *influential factor* was “liver” (515/3409 [15.12%] searches/100,000 inhabitants), followed by “stress” (365/3409 [10.72%] searches/100,000 inhabitants) and “allergy” (354/3409 [10.39%] searches per 100,000 inhabitants). Of all categories, *questions on pruritus* was the category with the fewest number of searches: 1186 searches per 100,000 inhabitants. The proportion ranged from 5.0% to 6.4% among the cities, with the lowest proportion recorded in Dresden and the highest in Dortmund (see Table 1 and 2).

The vast majority of categories were negatively correlated with each other. The categories *conditions*, *influential factors*, and *general* showed a negative correlation with all other categories. Compared to that, a positive correlation was detected between the categories *questions on pruritus* and *causes* (r=.12, *P*<.001) as well as *treatment* (r=.34, *P*<.001).

### Time Course of Search Behavior

**Figure 3:**
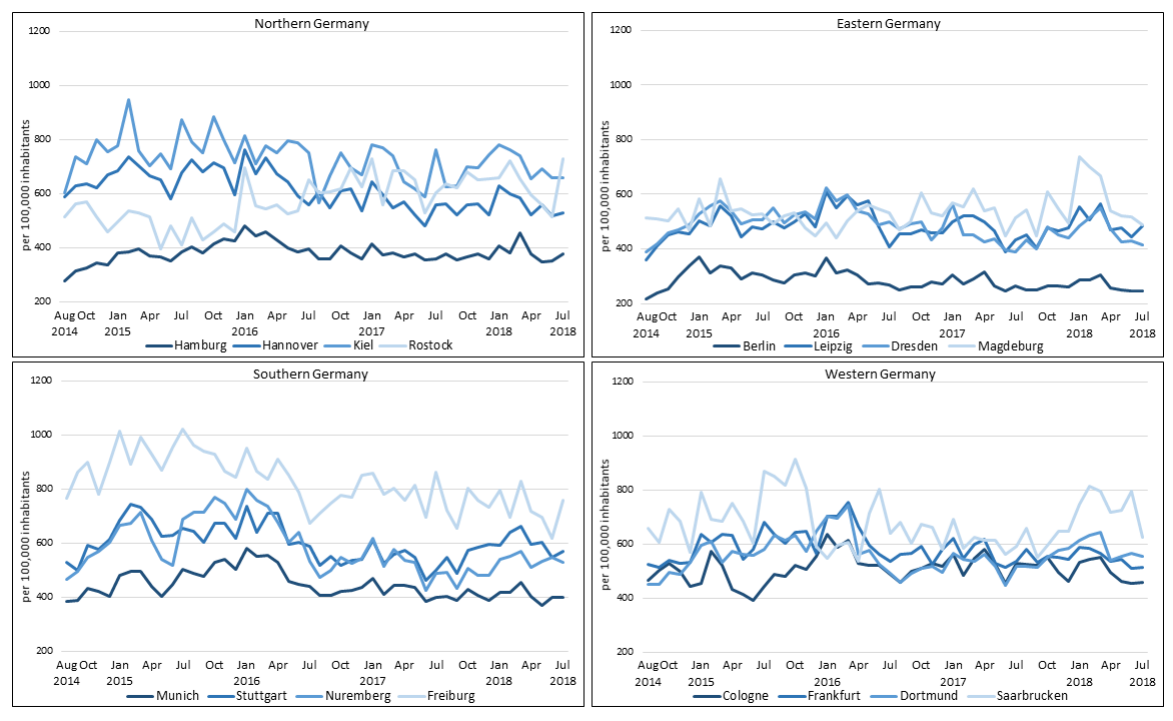
Trends in Google search volume per 100,000 inhabitants for pruritus-related keywords in 16 German cities from August 2014 to July 2018.

The average monthly number of searches was 452 per 100,000 inhabitants, with the greatest number occurring in Freiburg (n=823) and the smallest in Berlin (n=284). While the monthly number of searches per capita remained relatively stable in Berlin (range 239-370: 131) and Hamburg (range 316-482: 166), high seasonal ranges were observed in Kiel (range 566-946: 390) and Freiburg (range 620-1019: 399). Interestingly, a major decrease in search queries was observed in Saarbrucken from October 2015 to April 2016, whereas it was increased in all other cities in Western Germany. Except for Frankfurt, Dortmund, Freiburg, and Saarbrucken, the vast majority of cities had the highest relative number of search queries during the winter (ie, December to February). Across the four aberrant cities, the greatest numbers of searches were observed in March 2016 in Frankfurt (n=754), March 2016 in Dortmund (n=743), July 2015 in Freiburg (n=1019), and October 2015 in Saarbrucken (n=913). Overall, the highest number of relative searches occurred in January 2016 (n=562) and the lowest occurred in August 2014 (n=378) (see Figure 3).

**Table 2:** The five most-searched-for terms within each category across all examined cities, expressed as search queries per 100,000 inhabitants.

Category and search terms		n^a^ (%)
**Conditions (N=1896)**		
	Most common: During night-time	666 (35.12)
	Second-most common: During the evening	499 (26.30)
	Third-most common: During pregnancy	217 (11.46)
	Fourth-most common: Heat-induced	94 (4.97)
	Fifth-most common: During winter	87 (4.60)
**Influencing factors (N=3409)**		
	Most common: Liver	515 (15.12)
	Second-most common: Stress	365 (10.72)
	Third-most common: Allergies	354 (10.39)
	Fourth-most common: Diabetes	331 (9.70)
	Fifth-most common: Dry skin	319 (9.34)
**Localization (N=9759)**		
	Most common: Whole body	3375 (34.59)
	Second-most common: Legs	1516 (15.53)
	Third-most common: Anal or genital area	1127 (11.55)
	Fourth-most common: Hands	877 (8.98)
	Fifth-most common: Head	791 (8.11)
**Pruritus descriptors (N=2566)**		
	Most common: Strong	812 (31.65)
	Second-most common: Constant	457 (17.81)
	Third-most common: Sudden	392 (15.26)
	Fourth-most common: Extreme	286 (11.16)
	Fifth-most common: Burning	142 (5.54)
**Questions on pruritus (N=1186)**		
	Most common: What helps against pruritus?	120 (10.12)
	Second-most common: What helps against pruritus on the whole body?	45 (3.79)
	Third-most common: Why does the skin develop pruritus?	45 (3.79)
	Fourth-most common: What helps against itchy skin?	41 (3.45)
	Fifth-most common: How does pruritus occur?	38 (3.20)
**Treatment (N=2392)**		
	Most common: General	1205 (50.36)
	Second-most common: Ointment	290 (12.14)
	Third-most common: Cream	271 (11.31)
	Fourth-most common: Drugs	204 (8.54)
	Fifth-most common: Home remedies	179 (7.48)

^a^Number of searches/100,000 inhabitants.

### Supply of Dermatologists and General Practitioners

A high correlation between the number of search queries per 100,000 inhabitants and the supply of dermatologists (r=.56, *P*=.02) was identified (see Figure 1). This correlation was stronger than the correlation with the supply of GPs (r=.34, *P*=.19). When search terms that did not explicitly include pruritus (eg, “psoriasis” or “atopic eczema”) were also included in the analyses, the correlation was .51 (*P*=.05) for the supply of dermatologists and .50 (*P*=.05) for that of GPs.

### Inhabitants’ Demographics

The number of search queries per 100,000 inhabitants was negatively correlated with the respective inhabitants’ mean age (r=-.12, *P*=.67) and the proportion of nonnative Germans (r=-.05, *P*=.85). In contrast, the proportion of female searchers showed a positive association (r=.33, *P*=.21). However, none of these correlations were significant.

### Environmental Factors

Overall, a negative correlation was observed between searches and temperature (r=-.14, *P*<.001). This correlation was especially high in Berlin and Leipzig (r=-.59, *P*<.001 and r=-.52, *P*<.001, respectively). In contrast to temperature, monthly relative humidity was positively correlated with the searches, indicating that the number of search queries increased with higher humidity (r=.15, *P*<.001). The most significant correlations with humidity were detected in Berlin (r=.32, *P*=.03), Cologne (r=.33, *P*=.02), and Kiel (r=.35, *P*=.02) (see Table 3).

Overall, the monthly mean daily temperature had the greatest effect on the number of search queries per 100,000 inhabitants, with lower temperatures resulting in a higher number of search queries (beta -7.94, 95% CI -10.74 to -5.15). This effect was particularly high in Eastern Germany (beta -11.74, 95% CI -15.94 to -7.54) and Western Germany (beta -9.23, 95% CI -12.5 to 5.92). The level of PM10 was also associated with a highly negative effect (beta -5.13, 95% CI -7.04 to -3.22), whereas relative humidity (beta 4.72, 95% CI 2.70 to 6.75) and the duration of sunshine (beta 0.66, 95% CI 0.36 to 0.97) showed a positive effect. However, when analyzing these influences with respect to different regions, PM10 was found to exert a highly positive effect in Northern Germany (beta 6.42, 95% CI 2.41 to 10.43). In contrast, a negative effect was observed in all the other regions: Eastern Germany (beta -7.33, 95% CI -10.42 to -4.25), Southern Germany (beta -3.50, 95% CI -6.65 to -0.35), and Western Germany (beta -8.14, 95% CI -10.75 to -5.53) (see Table 4).

**Table 3:** Correlation between the number of search queries and selected environmental factors across 16 German cities from August 2014 to July 2018.

Cities	Monthly temperature (°C)			Monthly humidity (%)			Monthly sunshine duration (hours)			Monthly PM10^a^ (µg/m^3^)		
	Mean (SD)	r^b^	P	Mean (SD)	r	P	Mean (SD)	r	P	Mean (SD)	r	P
Berlin	11.0 (7.0)	-.59	<.001	73.0 (9.9)	.32	.03	140.1 (81.0)	-.41	.003	23.9 (5.9)	.28	.06
Hamburg	10.1 (9.2)	-.44	.002	80.4 (7.1)	.18	.22	131.5 (76.8)	-.33	.02	20.0 (4.5)	.11	.48
Munich	10.7 (7.1)	-.33	.02	72.8 (7.5)	.07	.63	146.9 (74.0)	-.20	.18	20.5 (6.8)	.27	.06
Cologne	12.0 (6.1)	-.47	.001	74.9 (8.3)	.33	.02	125.2 (66.2)	-.41	.004	23.3 (4.1)	.13	.37
Frankfurt	11.4 (6.8)	-.36	.01	74.2 (10.1)	.04	.80	133.3 (77.6)	-.18	.24	21.3 (5.1)	.06	.70
Stuttgart	11.3 (6.8)	-.41	.003	72.6 (8.3)	.01	.97	148.0 (75.3)	-.29	.046	19.9 (6.7)	.12	.40
Dortmund	11.0 (6.8)	-.47	.001	77.1 (6.4)	.10	.50	128.6 (63.2)	-.28	.06	23.0 (4.9)	.004	.98
Leipzig	10.6 (6.8)	-.52	<.001	75.9 (8.0)	.09	.56	137.8 (72.9)	-.29	.049	21.0 (5.8)	.38	.007
Dresden	11.1 (6.9)	-.48	.001	73.2 (7.8)	.23	.12	136.1 (69.8)	-.24	.10	21.1 (5.7)	.50	<.001
Hannover	10.5 (6.2)	-.38	.008	78.4 (7.5)	.21	.15	127.2 (72.3)	-.27	.07	19.1 (4.2)	.35	.01
Nuremberg	10.4 (7.0)	-.31	.03	75.9 (9.6)	.12	.42	144.1 (81.1)	-.32	.03	24.0 (8.4)	.33	.02
Kiel	10.0 (5.9)	-.38	.009	82.5 (5.7)	.35	.02	135.0 (90.7)	-.37	.009	21.9 (5.8)	.54	<.001
Magdeburg	10.8 (6.8)	-.33	.02	75.9 (8.7)	.11	.45	142.5 (78.4)	-.14	.36	20.3 (4.8)	.04	.80
Freiburg	11.4 (6.7)	-.19	.20	75.9 (7.3)	.06	.67	149.9 (76.3)	-.14	.36	16.0 (4.8)	.30	.04
Rostock	10.2 (6.1)	-.22	.13	79.0 (5.2)	.22	.13	150.8 (90.6)	-.18	.21	19.8 (5.1)	.04	.79
Saarbrucken	11.2 (6.5)	.08	.57	78.2 (9.3)	-.22	.14	134.4 (80.7)	.04	.80	18.0 (4.2)	-.05	.76
All cities	10.9 (6.5)	-.14	<.001	76.2 (8.4)	.15	<.001	138.2 (76.7)	-.09	.01	20.8 (5.9)	-.08	.04

^a^PM10: particulate matter 10 micrometers or less in diameter.

^b^Pearson’s correlation coefficient (r) was used to assess the correlation between the number of search queries and selected environmental factors within the city.

**Table 4:** Results of the linear regression using forward selection to assess the relationship between environmental factors and number of search queries per 100,000 inhabitants.

Covariates	Linear regression results for each German region, OR^a^ (95% CI)				
	Overall	Northern Germany	Eastern Germany	Southern Germany	Western Germany
PM10^b^	-5.13 (-7.04 to -3.22)	6.42 (2.41 to 10.43)	-7.33 (-10.42 to -4.25)	-3.50 (-6.65 to -0.35)	-8.14 (-10.75 to -5.54)
Temperature	-7.94 (-10.74 to -5.15)	N/A^c^	-11.74 (-15.94 to -7.54)	N/A	-9.23 (-12.5 to 5.92)
Sunshine duration	0.66 (0.36 to 0.97)	N/A	1.25 (0.71 to 1.79)	N/A	0.40 (0.13 to 0.67)
Humidity	4.72 (2.70 to 6.75)	N/A	7.07 (3.41 to 10.72)	3.58 (0.80 to 6.36)	N/A

^a^OR: odds ratio.

^b^PM10: particulate matter 10 micrometers or less in diameter.

^c^Not applicable; since a linear regression using forward selection was performed, some of the variables were not significant within each region.

## Discussion

### Principal Findings

From August 2014 to July 2018, 2,851,290 Google searches were performed for pruritus in the 16 examined German cities. Overall, the most common search terms were “pruritus,” “anal pruritus,” “pruritus on the whole body,” and “itchy skin.” The analyses showed that Berlin, Hamburg, and Munich, Germany’s three most-populated cities, had a comprehensively lower number of search queries per 100,000 inhabitants compared with smaller cities like Saarbrucken, Kiel, and Freiburg. In almost all cities, the highest number of searches were observed in the winter, with most occurring in January 2016 and the least number of searches occurring in August 2014.

There is some evidence that Google search analyses are an effective and inexpensive tool for assessing disease trends, such as seasonal variation in multiple medical fields [27,29,43]. Previous studies using Google Trends data demonstrated correlations with various sources of data, such as coronary heart disease epidemiology [44], cancer incidence and mortality rates [29], and cases of Ebola virus disease [45]. Each of the studies showed a positive correlation, which suggested that this is an alternative solution for disease surveillance [43]. This study investigated a possible correlation between search volume and the supply of physicians. The results indicated that the number of search queries showed a higher correlation with the supply of dermatologists (r=.56) than with the supply of GPs (r=.34). This was also the case when considering search terms, such as “psoriasis” or “atopic eczema.” One explanation for this discrepancy could be that, comparable to the diagnosis of xerosis [36], people consulting a dermatologist might receive a specific diagnosis for their pruritus and, therefore, may have performed explicit searches for pruritus. However, people seeing GPs might receive other less-specific diagnoses. Otherwise, this could indicate that, in spite of a high supply of physicians, there is still a definite need for medical care.

This study also revealed that the number of searches was correlated with climate. Interestingly, the effect of weather data differed within the regions. For example, in Northern Germany, only PM10 was found to have a significant effect on search behavior, while in Eastern Germany, all environmental factors examined were significant. Additionally, PM10 had a positive effect on the number of search queries in Northern Germany, which was contrary to all other regions. Although this study points to a negative effect from PM10, a positive effect, as it was observed in Northern Germany, is feasible since previous studies reported an association with pruritus or eczema [46]. Humidity and duration of sunshine were found to have a consistent positive effect across all regions. Temperature, in particular, was found to have a great effect on the number of search queries. It was found that a 1°C decrease in average monthly temperature correlated with around 8 more search queries per 100,000 inhabitants, with a greater effect observed in Eastern and Western Germany. Previous studies already reported that temperature influences the occurrence of pruritus [33,35]; for example, pruritus caused by xerosis is more common in colder months [36,47]. Similar to other German studies [30,31], we found an overall negative correlation between temperature and search volume across all cities during the entire study period (r=-.14). A study in the United States and the United Kingdom, however, reported contrary results (r_s_=.42 and r_s_=.27, respectively) [48]. Since no evidence was found that the United States or the United Kingdom generally have a warmer climate, search behavior has to be influenced by several other factors, for example, populations’ demographics, health campaigns, or medial visibility of diseases [23,49,50]. Despite an overall negative correlation with temperature, there were a few cities with a strikingly high number of searches in warmer months: Kiel, Freiburg, and Saarbrucken in July 2015. However, no considerable search-related differences according to temperature, humidity, PM10, or cumulative sunshine duration were found in comparison with other years. Thus, it confirms that there must be additional variables (eg, prevalence of allergies and pollen season) that influence search behaviors. This seems to be supported by the fact that a strong decline in search volume was observed in Saarbrucken from October 2015 to April 2016, while there were increases in Cologne, Dortmund, and Frankfurt, even though no significant differences in environmental factors were identified.

Besides examining disease trends, Google data can also be used for gaining insights into people’s health information-seeking behaviors [23,30,31]. Previous studies examining Google search volumes of pruritus-related keywords across Germany showed that the vast majority of searches (72.0%-72.6%) focused on *influential factors* [30,31], whereas most of the searches we found were related to *localization* (43.4%-47.5%) and only a small proportion were focused on *influential factors* (13.2%-16.6%). These differences might have occurred because this study only considered urban populations and search terms that explicitly included words or phrases related to pruritus, while other studies considered a larger population and additional keywords such as “psoriasis” and “atopic eczema.” Apart from this discrepancy, our results are mainly consistent with others in the literature. A differentiated consideration of the cities further enables the identification of general differences in medical needs or for a specific condition. When setting the search volume in relation to the respective inhabitants, we found that smaller cities were more likely to have a higher number of search queries. The highest relative search volume was observed in Freiburg, which might be somewhat influenced by the highest proportion of female inhabitants and lowest mean age in comparison to all other cities [39,41]. The results also indicated that people living in Berlin searched less frequently for information, and those who used Google were more likely to use unspecific keywords, which is expressed by the highest proportion of general keywords. At the same time, people living in Berlin or Munich seemed to have an especially high interest in anal pruritus, as 20.57% and 20.11%, respectively, of all *localization*-related searches focused on anal pruritus (overall 11.55%). If prevention campaigns could be adapted to regional differences, they might help to better address people’s therapeutic needs.

### Limitations

There are some limitations to this study that should be discussed. Google analyses are restricted to people who have access to the Internet and use Google as a search engine. In general, younger people use the Internet more frequently [24]. Since the Google AdWords Keyword Planner does not provide information on users’ general demographics, no statement about the examined population can be made. Although no clear correlation between the inhabitants’ mean age, percentage of female inhabitants, or percentage of nonnative Germans was found in this study, the study results might be somewhat influenced by them. On the one hand, this might have led to an underestimation of the pruritus burden, which is actually more prevalent among older people [9]. On the other hand, differences in the number of searches could result from the age distribution of Google users across the cities. Moreover, the search volume could have been influenced by different proportions of nonnative German speakers within the cities, as the study setting was limited to people whose language of preference was German, with respect to Google use and German search terms. A further limitation was that Google provides automatic completion of search terms, which might bias people’s search behavior.

### Conclusions

By taking into account the study objective and results, analyses of Google data are extremely useful for medical care since the public’s search behavior, including interest and frequency, could be investigated. Moreover, the study found that, for example, the number of search queries was negatively correlated with temperature and PM10, whereas it was positively correlated with humidity and sunshine duration. Considering this information, the analyses seemed to be valuable in forecasting higher public interest and need when weather is changing. In addition to this study, future studies could focus on age- or sex-specific aspects of dermatologic conditions to better target population-specific health care needs by implementing specifically adapted public health campaigns.

## Appendix

Multimedia Appendix 1 List of keywords and number of times searched.

## Abbreviations

ANOVA: one-way analysis of variance
GP: general practitioner
OR: odds ratio
PM10: particulate matter 10 micrometers or less in diameter
