# Supplementary material for: Influence of Climate on Google Internet Searches for Pruritus Across 16 German Cities: Retrospective Analysis
Source: J Med Internet Res. 2019 Jul 12;21(7):e13739. doi: 10.2196/13739 (PMC6659391; doi:10.2196/13739)
Supplement: Multimedia Appendix 1 [file jmir_v21i7e13739_app1.pdf]

Multimedia Appendix 1. *Most frequently searched pruritus-related keywords in 16 German cities from August 2014 to July 2018.*

Ranking	Keyword	Search volume
1	Juckreiz	115,680
2	Jucken am After	62,720
3	Juckreiz am ganzen Körper	56,660
4	Juckende Haut	53,480
5	Juckreiz After	39,670
6	Jucken im Ohr	34,130
7	Hautjucken	29,660
8	Juckende Beine	28,520
9	Jucken	26,710
10	Salbe gegen Juckreiz	21,470
11	Haut juckt	20,720
12	Juckreiz am Körper	19,020
13	Jucken im Genitalbereich	18,910
14	Juckreiz Schwangerschaft	16,190
15	Juckende Füße	16,140
16	Juckende Brust	13,450
17	Juckreiz Kopfhaut	12,970
18	Creme gegen Juckreiz	12,900
19	Hausmittel gegen Juckreiz	12,140
20	Juckende Hände	12,050
21	Beine jucken	11,440
22	Juckreiz an den beinen	11,160
23	Was hilft gegen Juckreiz	10,860
24	Juckende Schienbeine	10,030
25	Juckreiz rücken	10,020
26	Ganzer Körper juckt	9860
27	Juckreiz Leber	9770
28	Analer Juckreiz	9470
29	Hände jucken	9350
30	Juckreiz am ganzen Körper nachts	9220
31	Trockene juckende haut	8950
32	Hautjucken Ursachen	8780
33	Juckreiz am ganzen Körper ohne Ausschlag	8650
34	Starker Juckreiz	8560
35	Mittel gegen Juckreiz	8500
36	Juckreiz Hausmittel	8200
37	starker Juckreiz am ganzen Körper	8140
38	Juckreiz im Gesicht	8010
39	Juckreiz am hals	8000
40	Plötzlicher Juckreiz am ganzen Körper	7850
41	Gegen Juckreiz	7760
42	Jucken im Analbereich	7590

43	Medikamente gegen Juckreiz	7400
44	Juckreiz lindern	7390
45	Juckreiz Genitalbereich	7350
46	Juckende Unterschenkel	7100
47	Juckreiz am ganzen Körper Leber	7070
48	Juckreiz ganzer Körper	7050
49	Trockene Haut Juckreiz	6990
50	Juckreiz Füße	6880
